# Supplementary material for: Strain Improvement and Strain Maintenance Revisited. The Use of Actinoplanes teichomyceticus ATCC 31121 Protoplasts in the Identification of Candidates for Enhanced Teicoplanin Production
Source: Antibiotics (Basel). 2021 Dec 27;11(1):24. doi: 10.3390/antibiotics11010024 (PMC8773182; doi:10.3390/antibiotics11010024)
Supplement: Supplementary file 1 [file antibiotics-11-00024-s001.zip › antibiotics-1444163-supplementary.pdf]

antibiotics MDPI

Supplementary material

Table S1. Elution order and relative retention time of the teicoplanin product according to Ph. Eur.

Name of component	Elution order (Ph. Eur.)	Relative retention time (Ph. Eur.)*
Teicoplanin A3-1	1	0.43
Teicoplanin A2-1	2	0.93
Teicoplanin A2-2	3	1.00
Teicoplanin A2-3	4	1.04
Teicoplanin A2-4	5	1.12
Teicoplanin A2-5	6	1.14

*the time of elution is relative to Teicoplanin A2-2

Table S2. Specifications of the teicoplanin product according to J. P. and Ph. Eur.

Name of component	Limits adopted by J.P. for content of each component of the complex	Limits adopted by Ph. Eur. for content of each component of the complex
Teicoplanin A3	NMT 15.0%	NMT 15.0%
Teicoplanin A2 group*	NLT 80.0%	NLT 80%
Teicoplanin A2-1	NA	NMT 20%
Teicoplanin A2-2	NA	NLT 35% and NMT 55%
Teicoplanin A2-3	NA	NMT 20%
Teicoplanin A2-4	NA	NMT 20%
Teicoplanin A2-5	NA	NMT 20%
impurities	NMT 5%	NMT 5%

* As group the sum of all the A2 factors is indicated

NLT: Not Less Than

NMT: Not More Than

NA: Not Applicable

Table S3. Summary statistics of fermentations to produce teicoplanin performed using hyphae- and protoplasts-derived clones.

**Teicoplanin complex Factors in production from Hyphae**	**N**	**Mean**	**St. Dev.**	**Min**	**Pctl(25)**	**Pctl(75)**	**Max**
TA2-1	49	5.242	1.372	2.239	4.251	6.416	8.287
TA2-2	49	88.130	36.214	22.084	68.754	114.649	161.687
TA2-3	49	43.424	19.839	11.953	28.195	58.074	96.498
TA2-4	49	43.439	16.152	8.900	30.176	51.000	85.040
TA2-5	49	38.766	14.025	4.914	28.676	46.366	75.849
Complex Sum	49	219.111	65.898	110.983	169.705	274.221	373.695
**Teicoplanin complex Factors in production from Protoplasts**	**N**	**Mean**	**St. Dev.**	**Min**	**Pctl(25)**	**Pctl(75)**	**Max**
TA2-1	49	8.876	8.385	0	4.8	8.7	32
TA2-2	49	135.479	60.614	8.695	110.038	151.891	331.047
TA2-3	49	50.974	29.381	0	23.7	78.4	103
TA2-4	49	46.723	23.268	0	32.9	66.5	89
TA2-5	49	40.567	19.119	0	28.0	52.0	87
Complex Sum	49	282.619	110.026	12.562	215.429	348.970	507.852

Table S4. Teicoplanin production and complex composition in hyphae-derived clones.

Clone	TA2-1 (%)	TA2-2 (%)	TA2-3 (%)	TA2-4 (%)	TA2-5 (%)	Teicoplanin (mg/L)
**Ph. Eur.**	**< 20**	**> 35 and < 55**	**< 20**	**< 20**	**< 20**	**Not applicable**
H1	4,2	16,7	30,3	30,2	18,7	138,4
H2	3,4	10,8	32,2	37,6	16,0	226,2
H3	4,4	13,3	34,2	31,5	16,5	169,7
H4	3,8	15,4	32,0	31,8	16,9	169,2
H5	4,0	13,8	34,1	31,3	16,7	160,4
H6	2,1	41,7	14,5	21,3	20,4	201,1
H7	2,0	41,8	18,5	22,2	15,4	327,9
H8	1,9	39,5	18,6	21,2	18,8	218,5
H9	1,9	41,1	14,2	21,6	21,2	199,2
H10	2,2	37,7	19,8	20,1	20,2	197,3
H11	2,2	39,2	17,9	20,7	20,2	274,2
H12	2,3	39,0	17,4	19,7	21,5	258,3
H13	2,9	46,6	12,6	15,7	22,2	172,2
H14	2,0	44,5	14,9	19,7	18,8	128,6
H15	2,1	46,3	18,7	13,8	19,2	185,7
H16	2,1	49,2	16,5	16,2	16,1	312,0
H17	1,6	51,0	20,8	11,9	14,6	316,9
H18	2,1	57,9	9,2	14,3	16,5	240,0
H19	1,8	28,8	21,8	21,9	25,7	238,4
H20	2,8	56,3	8,8	14,2	18,0	178,2
H21	1,6	38,1	24,1	19,4	16,8	295,7
H22	4,3	71,8	10,8	8,7	4,4	111,0
H23	2,7	52,4	11,9	18,3	14,8	160,9
H24	2,6	43,1	19,8	18,1	16,3	251,1
H25	1,7	44,1	21,7	17,1	15,3	289,4
H26	1,7	44,1	21,7	17,1	15,3	289,3
H27	2,5	43,5	19,0	18,9	16,1	202,5
H28	2,9	45,8	12,4	20,7	18,2	189,7
H29	2,9	54,3	20,5	12,7	9,6	224,4
H30	3,5	51,4	9,6	19,0	16,5	188,7
H31	2,3	47,9	15,1	18,3	16,4	242,1
H32	1,8	41,1	24,3	18,0	14,8	334,1
H33	1,8	42,8	22,7	17,6	15,2	280,9
H34	2,2	45,5	20,7	17,6	14,0	263,8
H35	5,0	67,6	14,7	6,5	6,3	137,9
H36	3,5	36,6	16,1	23,7	20,1	188,6
H37	2,8	36,5	20,4	20,2	20,0	196,8
H38	2,4	46,6	14,5	18,8	17,8	259,5
H39	1,6	30,7	25,8	21,6	20,3	373,7
H40	2,4	38,3	19,0	21,9	18,3	170,3
H41	1,9	40,7	20,5	19,6	17,3	125,9
H42	3,6	36,2	17,2	22,1	20,9	133,2
H43	2,8	36,5	17,8	22,2	20,7	135,9
H44	2,5	37,3	21,3	20,2	18,7	132,7
H45	2,1	34,5	17,0	24,3	22,2	191,0
H46	2,8	23,8	25,2	25,4	22,8	294,8
H48	1,4	44,8	18,1	17,4	18,3	158,0
H49	2,6	24,0	25,3	25,2	22,8	294,8
H50	1,3	51,3	19,4	12,4	15,5	307,5

Table S5. Teicoplanin production and complex composition in protoplast-derived clones.

Clone	TA2-1 (%)	TA2-2 (%)	TA2-3 (%)	TA2-4 (%)	TA2-5 (%)	Teicoplanin (mg/L)
**Ph. Eur.**	**< 20**	**> 35 and < 55**	**< 20**	**< 20**	**< 20**	**Not applicable**
P31	4,1	42,0	21,6	17,0	15,2	280,9
P32	0,0	61,1	10,0	15,1	13,7	218,2
P33	9,5	39,8	19,5	18,2	13,0	296,7
P34	0,0	100,0	0,0	0,0	0,0	12,6
P35	0,0	57,4	12,2	16,9	13,6	215,4
P36	0,0	40,7	18,6	23,2	17,5	268,9
P37	0,0	55,8	12,3	17,7	14,2	285,8
P38	2,8	44,9	15,2	21,0	16,1	188,8
P39	3,3	76,2	8,8	5,5	6,3	233,8
P40	0,0	53,9	16,9	17,8	11,5	209,1
P41	2,1	36,0	25,2	20,1	16,6	349,0
P42	7,3	42,7	24,9	13,9	11,1	320,2
P43	7,7	50,5	17,2	14,2	10,3	271,5
P44	3,3	42,3	23,0	17,7	13,8	359,1
P45	7,7	49,2	19,4	14,5	9,1	234,1
P46	6,5	35,0	26,5	17,5	14,4	387,2
P47	7,9	45,9	21,0	14,1	11,1	403,3
P48	11,5	45,9	18,8	14,4	9,4	265,3
P49	0,7	60,8	16,8	10,5	11,2	463,9
P50	7,7	42,7	22,3	15,8	11,6	382,6
P51	2,8	57,4	18,4	9,2	12,2	401,8
P52	2,9	65,6	10,0	9,7	11,8	238,1
P53	3,6	70,6	7,9	7,6	10,3	188,4
P54	4,0	63,5	8,1	14,4	9,9	68,7
P55	2,8	52,2	9,9	14,8	20,3	192,4
P56	2,1	67,8	7,0	10,4	12,7	297,8
P57	2,5	53,2	3,3	21,9	19,0	302,5
P58	1,8	39,1	25,3	18,7	15,1	388,8
P60	1,4	37,0	20,9	19,4	21,2	410,0
P61	1,8	35,0	26,5	21,2	15,5	314,0
P62	1,6	36,1	18,8	22,3	21,1	398,6
P63	1,8	36,1	25,0	20,8	16,3	321,8
P64	2,4	40,4	21,0	19,8	16,5	198,3
P65	2,0	39,9	25,4	18,2	14,5	346,0
P66	1,4	34,4	17,9	24,6	21,8	345,8
P67	3,0	35,4	22,6	22,0	17,0	319,9
P68	4,6	66,9	12,1	7,6	8,8	46,9
P69	1,6	39,2	17,5	22,4	19,3	340,3
P70	4,1	67,1	9,4	7,8	11,6	183,0
P71	4,0	61,6	7,5	11,0	15,9	165,8
P72	1,8	60,1	13,4	11,1	13,7	199,7
P73	1,3	65,2	12,5	12,0	9,0	507,9
P74	1,4	55,4	18,7	14,6	9,8	485,6
P75	3,0	44,4	18,8	19,3	14,5	293,2
P76	2,5	35,5	15,9	26,4	19,7	281,4
P77	2,7	40,1	20,4	18,6	18,2	246,5
P78	2,4	31,2	24,9	22,1	19,4	314,5
P79	1,6	62,5	14,8	8,8	12,4	380,2
P80	6,4	36,2	14,7	19,6	23,1	24,0

Table S6. Correlation of teicoplanin factors production in hyphae-derived clones.

Parameter1	Parameter2	r	95% CI	t (47)	p
TA2-1	TA2-2	0.02	[-0.26, 0.30]	0.16	> .999
TA2-1	TA2-3	0.48	[0.23, 0.67]	3.72	0.004**
TA2-1	TA2-4	0.55	[0.31, 0.72]	4.46	< .001***
TA2-1	TA2-5	0.25	[-0.03, 0.50]	1.79	0.282
TA2-1	Complex Sum	0.37	[0.10, 0.59]	2.71	0.047*
TA2-2	TA2-3	0.26	[-0.02, 0.50]	1.85	0.282
TA2-2	TA2-4	0.06	[-0.23, 0.33]	0.39	> .999
TA2-2	TA2-5	0.39	[0.13, 0.61]	2.93	0.031*
TA2-2	Complex Sum	0.73	[0.56, 0.84]	7.22	< .001***
TA2-3	TA2-4	0.83	[0.72, 0.90]	10.20	< .001***
TA2-3	TA2-5	0.68	[0.50, 0.81]	6.44	< .001***
TA2-3	Complex Sum	0.81	[0.68, 0.89]	9.33	< .001***
TA2-4	TA2-5	0.77	[0.62, 0.86]	8.17	< .001***
TA2-4	Complex Sum	0.70	[0.53, 0.82]	6.78	< .001***
TA2-5	Complex Sum	0.83	[0.72, 0.90]	10.26	< .001***

p-value adjustment method: Holm (1979)

Observations: 49

Table S7. Correlation of teicoplanin factors production in protoplast-derived clones.

Parameter1	Parameter2	r	95% CI	t(47)	p
TA2-1	TA2-2	0.15	[-0.13, 0.42]	1.06	0.657
TA2-1	TA2-3	0.42	[0.15, 0.63]	3.15	0.020*
TA2-1	TA2-4	0.18	[-0.11, 0.44]	1.25	0.657
TA2-1	TA2-5	0.07	[-0.21, 0.35]	0.49	0.657
TA2-1	Complex.Sum	0.32	[0.04, 0.55]	2.33	0.105
TA2-2	TA2.3	0.44	[0.18, 0.64]	3.34	0.013*
TA22	TA2-4	0.33	[0.05, 0.56]	2.39	0.105
TA2-2	TA2-5	0.40	[0.13, 0.61]	2.98	0.027*
TA2-2	Complex.Sum	0.82	[0.70, 0.89]	9.76	< .001***
TA2-3	TA2-4	0.79	[0.66, 0.88]	8.96	< .001***
TA2-3	TA2-5	0.73	[0.56, 0.84]	7.29	< .001***
TA2-3	Complex.Sum	0.83	[0.72, 0.90]	10.39	< .001***
TA2-4	TA2-5	0.92	[0.87, 0.96]	16.42	< .001***
TA2-4	Complex.Sum	0.78	[0.64, 0.87]	8.51	< .001***
TA2-5	Complex.Sum	0.79	[0.65, 0.88]	8.80	< .001***

p-value adjustment method: Holm (1979)

Observations: 49

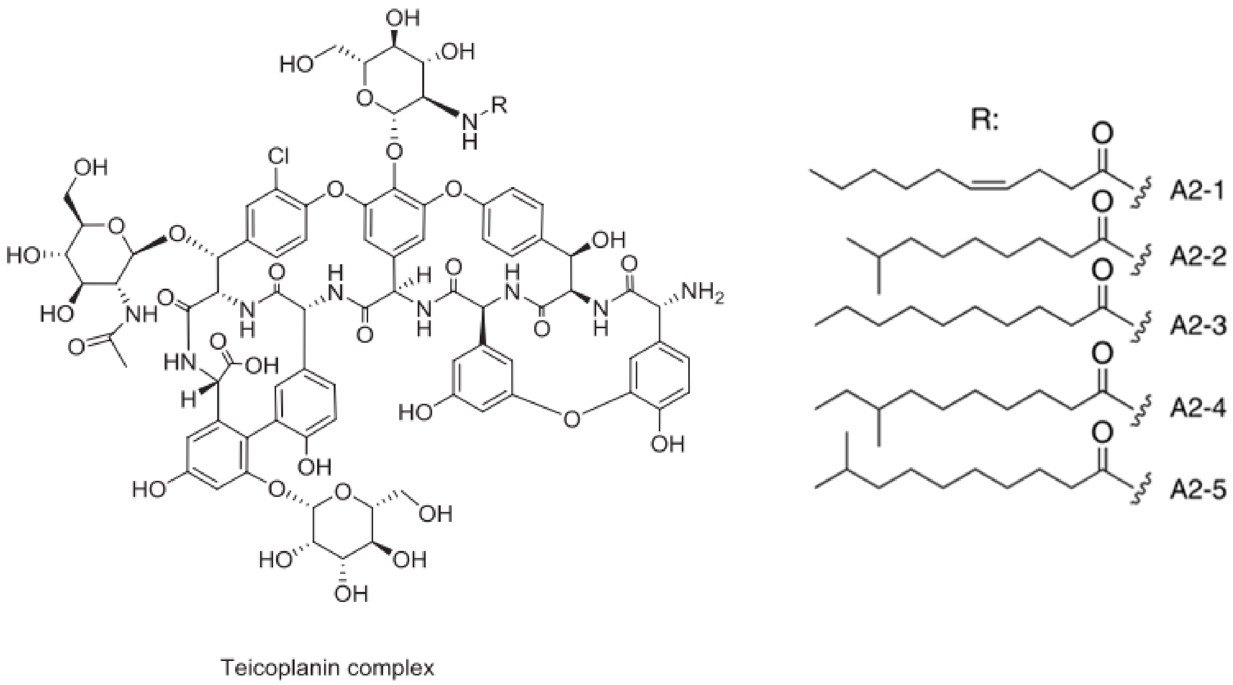

Figure S1. Teicoplanin chemical structure and differences among the factors of the teicoplanin complex.

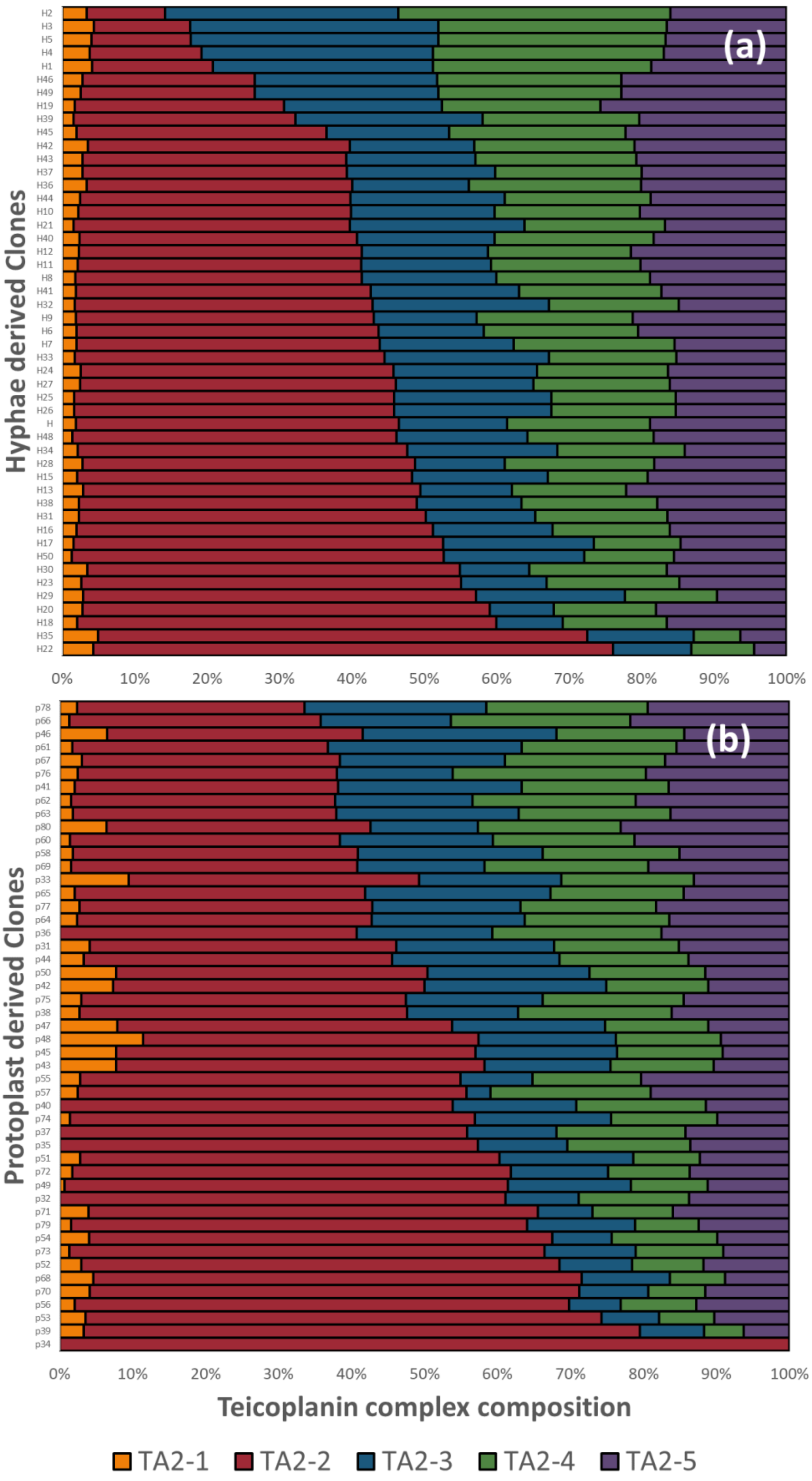

Figure S2. Distribution of teicoplanin complex factors in hyphae-derived (a) and protoplast-derived (b) fermentations.

antibiotics MDPI

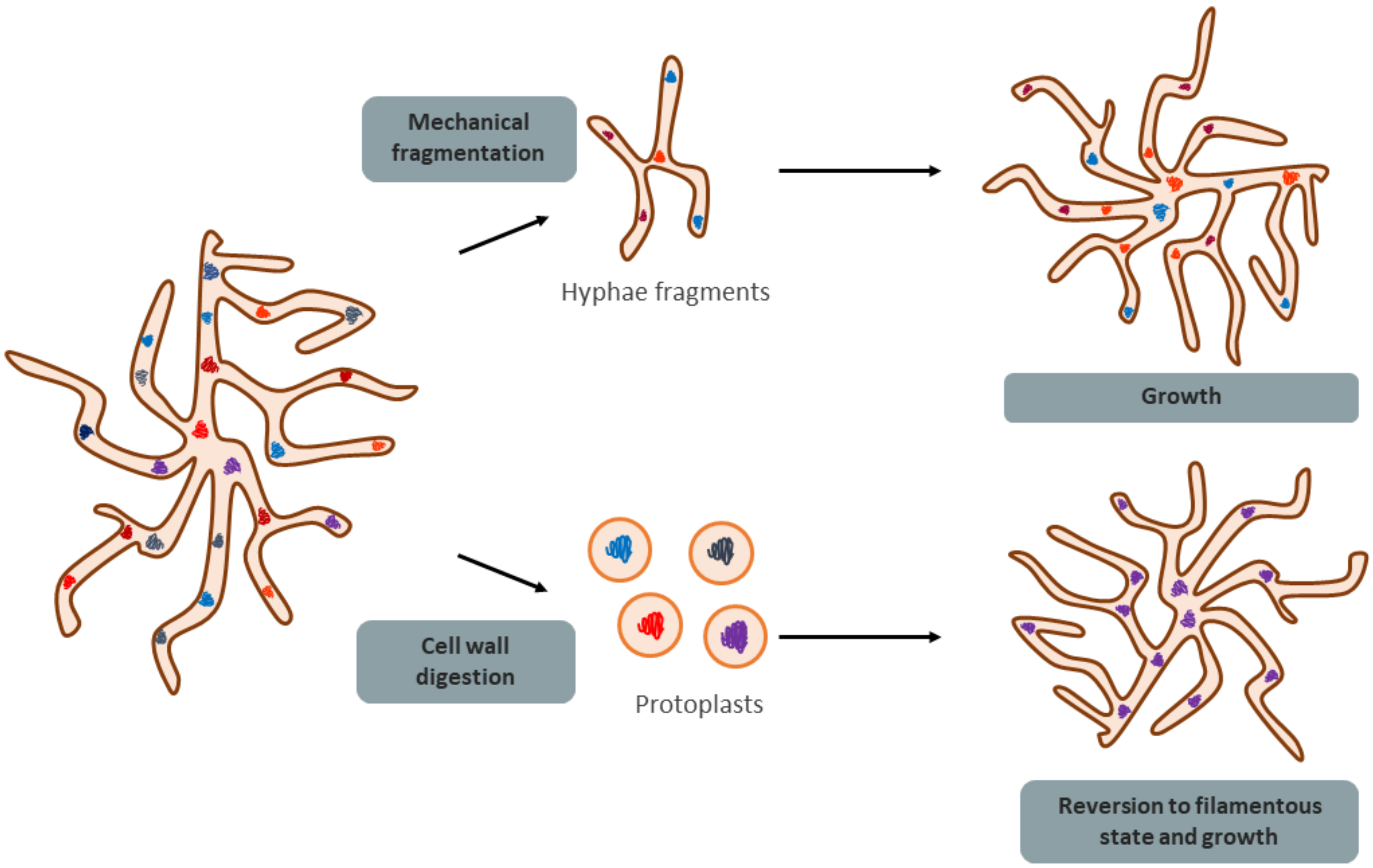

Figure S3. Representation of the technique used for the separation of genomes in filamentous actinomycetes and advantages over the simple mechanical fragmentation. From a conceptual point of view, the presence of more than one genome and/or mutations in the genomes embedded in protoplasts is not excluded.
